# Dental status, dental rehabilitation procedures, demographic and oncological data as potential risk factors for infected osteoradionecrosis of the lower jaw after radiotherapy for oral neoplasms: a retrospective evaluation

**DOI:** 10.1186/1748-717X-8-227

**Published:** 2013-10-02

**Authors:** Marcus Niewald, Jochen Fleckenstein, Kristina Mang, Henrik Holtmann, Wolfgang J Spitzer, Christian Rübe

**Affiliations:** 1 Department of Radiotherapy and Radiooncology, Saarland University Medical Center, Kirrberger Str. 1, D-66421 Homburg, Germany; 2 Dental Practice, Duisburg, Germany; 3 Department of Oral and Maxillofacial Surgery, Saarland University Medical Center, Kirrberger Str. 1, D-66421 Homburg, Germany

**Keywords:** Dental status, Dental rehabilitation procedures, Radiotherapy, Fractionation, Infected osteoradionecrosis

## Abstract

**Purpose:**

Retrospective evaluation of the dental status of patients with oral cancer before radiotherapy, the extent of dental rehabilitation procedures, demographic and radiotherapy data as potential risk factors for development of infected osteoradionecrosis of the lower jaw.

**Methods:**

A total of 90 patients who had undergone radiotherapy for oral cancer were included into this retrospective evaluation. None of them had distant metastases. After tumour surgery the patients were referred to an oral and maxillofacial surgeon for dental examination and the necessary dental rehabilitation procedures inclusive potential tooth extraction combined with primary soft tissue closure. Adjuvant radiotherapy was started after complete healing of the gingiva (> 7 days after potential extraction). The majority of patients (n = 74) was treated with conventionally fractionated radiotherapy with total doses ranging from 50-70Gy whereas further 16 patients received hyperfractionated radiotherapy up to 72Gy. The records of the clinical data were reviewed. Furthermore, questionnaires were mailed to the patients’ general practitioners and dentists in order to get more data concerning tumour status and osteoradionecrosis during follow-up.

**Results:**

The patients’ dental status before radiotherapy was generally poor. On average 10 teeth were present, six of them were regarded to remain conservable. Extensive dental rehabilitation procedures included a mean of 3.7 tooth extractions. Chronic periodontitis with severe attachment loss was found in 40%, dental biofilm in 56%. An infected osteoradionecrosis (IORN) grade II according to (Schwartz et al., Am J Clin Oncol 25:168-171, 2002) was diagnosed in 11 of the 90 patients (12%), mostly within the first 4 years after radiotherapy. We could not find significant prognostic factors for the occurrence of IORN, but a trendwise correlation with impaired dental status, rehabilitation procedures, fraction size and tumour outcome.

**Conclusion:**

The occurrence of IORN is an important long-term side effect of radiotherapy for oral cancers. From this data we only can conclude that a poor dental status, conventional fractionation and local tumour progression may enhance the risk of IORN which is in concordance with the literature.

## Background

The dental status of patients with neoplasms of the head and neck region is known to be more unfavourable compared to healthy persons. Some reasons may be the frequent abuse of nicotine and alcohol and limited dental hygiene. These factors may increase the risk for developing infected osteoradionecrosis (IORN) after radiotherapy as a typical long-term side effect [1]. To our knowledge, IORN can only be sufficiently avoided performing by extensive dental rehabilitation procedures including extraction of teeth. Nevertheless, IORN occurs in a frequency ranging from 0-22% [2]. Because of the necrosis of the gingiva, erosion and sequestration of the jaw bone and dentoalveolar abscess formation sufficient chewing and thus nutrition of the patient can be problematic and thus impair the quality of life of those patients by a large extent.

16 years ago we have published a retrospective evaluation of the frequency and risk factors for IORN [2]. The frequency of IORN in patients after conventionally fractionated radiotherapy (total dose 60-70Gy) was 8.6% while the same rate amounted to 22.9% in patients with hyperfractionated radiotherapy (total dose 82.8Gy). We concluded that this excessive occurrence of IORN in patients having been treated with a hyperfractionated irradiation regimen was probably caused by the high total dose and a too short interfraction interval.

In the presented analysis, we performed a second evaluation of frequency and risk factors for IORN in a totally different collective of patients having undergone radiotherapy in the years 1993–2001.

## Methods

Ninety patients with histologically proven squamous-cell cancers of the oral cavity treated in the years 1993–2001 were included into this retrospective evaluation. The majority had primary tumors, one a local recurrence but had not been irradiated before, none had distant metastases. The mean age at the beginning of radiotherapy was 57 years, the mean Karnofsky performance index 7.8. Sixty-four patients had undergone prior surgery, the remaining 24 had not.

Dental examination and treatment procedures were performed as early as possible with a minimal time interval of 7–10 days from the last procedure to the beginning of radiotherapy. All dental extractions were performed according to a written protocol under “special care” (primary tissue closure, perioperative antibiotics for 7–10 days beginning one day before surgery). In the nineties all patients were advised not to wear their dental prostheses up to 6–12 months after radiotherapy (today after complete healing of mucositis) [3,4].

After completion of all dental examination and rehabilitation procedures (Tab. 3), all patients underwent radiotherapy. 74 patients were treated in a conventionally fractionated manner applying total doses of 60Gy (n = 33) up to 70Gy (n = 23). Ten patients received 64Gy mainly due to compensation for holidays and accelerator breakdowns while the remaining 10 patients got 36-58Gy mostly due to deterioration of the general health status during radiotherapy or the withdrawal of informed consent by the patient. Sixteen patients were treated in a hyperfractionated manner with two daily single doses of 1.2Gy with an interfraction interval of more than 6 hours thus reaching a total dose of 72Gy (n = 11). The remaining four patients had total doses ranging from 58.8 to 76.8Gy due to the same reasons as stated above. Total doses were defined to the ICRU (International Commission on Radiation Units) 50 reference point. Volume data were available only for the last few patients so that these are considered here. The dose distributions were reviewed, the mandible was well within the 100% isodose.

After production of a fixation mask and the planning procedure based on a computerized tomography of the head and neck region, radiotherapy regularly was performed by laterolateral parallel opposing irregular portals formed by beam blocks or by a multileaf collimator using 4 – 6 MV photons of a linear accelerator. After a total dose ranging from 30-50Gy the spinal cord was spared by a dorsal field reduction. The resulting underdosage of the level V region was supplemented by applying lateral opposing electron portals there up to the primarily intended total dose.

In 17 patients not having been operated on before chemotherapy consisting of cis-platinum and 5-fluorouracil was applied simultaneously. Further demographical and oncological details are depicted in Table 1.

**Table 1:** Patients’ demographical and oncological data (n = 90)

**Item**	**Mean value**	**Minimum value**	**Maximum value**	**Remarks**
Age	56.97	21.1	84.0	Years
Karnofsky performance status	78%	50%	100%	
Follow-up	3.5	0	12.25	Years
**Item**	**Value (%)**			
T-stage				
T1	14 (16%)			
T2	33 (37%)			
T3	8 (9%)			
T4	35 (38%)			
N-stage				
N0	20 (22%)			
N1	24 (27%)			
N2	46 (51%)			
N3	0			
UICC stage				
I	7 (8%)			
II	6 (7%)			
III	11 (12%)			
IV	66 (73%)			
Pre-treatment				
None	27 (30%)			
Surgery	61 (70%)			
Surgery to the lower jaw				
Partial resection	16 (23%)			
Continuity resection	10 (14%)			
Chemotherapy	17 (19%)			
Radiotherapy – fractionation				
Conventional	74 (82%)			
Hyperfractionation	16 (18%)			
Fraction size				Gy/day
1 × 2.0	72 (80%)			
1 × 3.0	2 (2%)			
2 × 1.2	15 (17%)			
2 × 1.4	1 (1%)			
total dose – conventionally fractionated (n = 75)				Gy
30	1 (1%)			
36	1 (1%)			
50	7 (9%)			
58	2 (2%)			
60	31 (44%)			
64	9 (13%)			
70	23 (31%)			
Total dose – hyperfractionated (n = 15)				
58.8	1 (6%)			
70.8	1 (6%)			
72.0	12 (76%)			
72.8	1 (6%)			
76.8	1 (6%)			

During radiotherapy, the patients received oral care by the dental colleagues (inhouse). Fluoridation was performed according to dental advice. Splints were not used normally due to the experience that the majority of patients used them incorrectly and thus enhanced oral mucositis.

After radiotherapy, dental follow-up was performed by their local dentists. Consequently, detailed data about this phase are not available. Patients with IORN were referred to the Dept. of Oral and Maxillofacial Surgery for further treatment.

All patients’ records were reviewed. The dental status and the extent of dental rehabilitation procedures were extracted from the files in the Department of Oral and Maxillofacial Surgery. Furthermore, all x-rays (orthopantomograms) were re-examined. Infected osteoradionecrosis (IORN) was (at minimum) defined here as infected mucosal ulcers with eroded mandibular bone underneath (grade II according to Schwartz et al. [5]). The radiooncological data were extracted from the files in the Department of Radiotherapy and Radiooncology. Additionally, standardized questionnaires were mailed to the patients’ medical doctors, dentists and local authorities three times within the observation period in order to achieve additional data concerning tumor outcome, occurrence of IORN and general health status.

All data were entered into a medical database (MEDLOG, Parox Comp., Münster, Germany). Distributions and means were computed. For survival curves and failure curves (occurrence of IORN) the Kaplan Meier estimate was used. These curves were compared using the Mantel Haensel test.

Prognostic parameters for IORN were analyzed univariately by comparison of means and distributions in a group containing the patients with IORN compared to another group with the patients who never experienced IORN using the t-test, u-test and chi-square test in the appropriate variables. Multivariate search for independent prognostic factors was performed by logistic regression.

All patients had given their written informed consent to surgery, dental rehabilitation procedures and radiotherapy before treatment. The approval by the local ethics committee was dispensable due to the retrospective evaluation performed here. This research is in concordance with the Declaration of Helsinki.

## Results

### General remarks

Up to July 2013, 58 patients were dead with a mean follow-up of 2.4 [0–8.8] years. The patients known to be alive were seen irregularly, the most recent information resulted from questionnaires, nearly all patients were lost to follow-up after on average 7.4 [0–15] years.

### Dental findings before radiotherapy

The patients’ dental status was generally poor. On average, only 10 teeth were present (and thus 22 teeth missing) at the beginning of therapy in the oral cavity. Of those, on average 2.0 were carious, 1.5 loose (clinical grade II-III) and 1.4 deeply destroyed. As a result of the meticulous dental examination only a mean of six teeth was regarded to remain conservable on average. 11% of the patients showed chronic periodontitis with less to moderate attatchment loss while in 40% chronic periodontitis with severe attatchment loss was diagnosed.

Additionally, plaque was found frequently; 56% of the patients had dental biofilm and dental calculi and subgingival concrements, respectively. The general dental hygiene was classified separately by a dentist as poor in 55% of the patients. These findings are based on the data of 53 patients, further information was not available in the files. The detailed data are depicted in Table 2.

**Table 2:** Dental status before starting radiotherapy

**Teeth (n=)**	**Mean value**	**Minimum value**	**Maximum value**	**Data available from n patients**
Absent	22.0	0	32	89
Present	10.1	0	32	89
Carious	2.0	0	21	85
Destroyed	1.4	0	21	87
Loose	1.6	0	13	84
Root remainders	0.3	0	5	87
Devital	0.5	0	4	86
Roots – filled completely	0.2	0	2	84
Roots – filled incompletely	0.3	0	4	85
Apical periodontitis	0.3	0	4	86
Cysts	0.2	0	3	85
Retained	0.2	0	2	84
Conservative treatment possible	5.8	0	30	88
No conservative treatment possible	4.3	0	25	88
Filled	2.3	0	20	82
Not sufficiently filled teeth	0.8	0	11	82
Teeth with not sufficient crowns	0.2	0	2	82
**Item**	**Number of patients (%)**			**n=**
Chronic periodontitis with less to moderate attachment loss				
Localized	7 (8%)			
General	9 (11%)			85
Chronic periodontitis with severe attachment loss				87
Localized	12 (14%)			
General	35 (40%)			
Biofilm				53
None	1 (2%)			
Moderate	21 (42%)			
Intense	30 (56%)			
Dental calculi and subgingival concrements				53
None	1 (2%)			
Moderate	22 (42%)			
Intense	30 (56%)			
Dental hygiene				54
Good	3 (6%)			
Poor	21 (39%)			
Not sufficient	30 (55%)			

n=: data of n patients available.

Data on the use of dental prostheses are available for 65/90 patients. Partial prostheses were used by six patients in the upper jaw and by 22 patients in the lower jaw. Complete prostheses were used by 34 patients in the upper jaw and by 15 patients in the lower jaw. The patients had been counselled not to wear their prostheses during radiotherapy and 12 months afterwards.

### Dental rehabilitation procedures before and after radiotherapy

All patients were referred to the dentist and oromaxillofacial surgeon for dental and surgical treatment, 89/90 have been seen there. The dental rehabilitation procedures necessary before radiotherapy were very extensive: on average 3.7 teeth had to be extracted followed by primary soft tissue closure of the extraction alveoles. The extraction was followed up by an interval of at least 7–10 days using soft diet, valid antibiosis and prosthodontic abstention. The detailed data to this point are summarized in Table 3.

**Table 3:** Dental treatment procedures

**Teeth (n=)**	**Mean value**	**Minimum value**	**Maximum value**	**Data available from n patients**
Endodontic treatment	0.05	0	2	88
Removal of root remainders with primary tissue closure	0.2	0	5	88
Tooth extraction with primary tissue closure	3.7	0	22	89
Conserving treatment	0.6	0	6	88
Cystectomy	0.1	0	2	87
Healthy teeth remaining after dental rehabilitation	6.2	0	30	89
Edentulous after dental treatment	47 patients (52%)			

During follow-up after radiotherapy additional dental treatment procedures were necessary in 17 patients – the data are depicted in Table 4.

**Table 4:** Dental treatment after radiotherapy

**Treatment**	**Number of patients (%)**
None	73 (81%)
Conservative treatment	2 (2%)
Tooth extraction with primary tissue closure	14 (16%)
Tooth extraction with primary tissue closure and conservative treatment	1 (1%)

### Frequency, risk factors and therapy of infected osteoradionecrosis

11 patients (12%) were found to have developed infected osteoradionecrosis during follow-up. The one-year prevalence was 5%, the two- and three-year prevalence 15%.

All of them had been treated by conventionally fractionated radiotherapy applying doses of 50Gy (1 pat.), 60Gy (4 pats.), 64Gy (3 pats.), and 70Gy (3 pats.), respectively. Nine of these patients had undergone tumour resection, two had not. Additionally, two patients had undergone a partial resection of the lower jaw, further three patients a continuity resection of the lower jaw. In total, 9/64 patients (14%) having been operated on had IORN compared to only 8% in the non-surgical patients.

**Figure 1:**
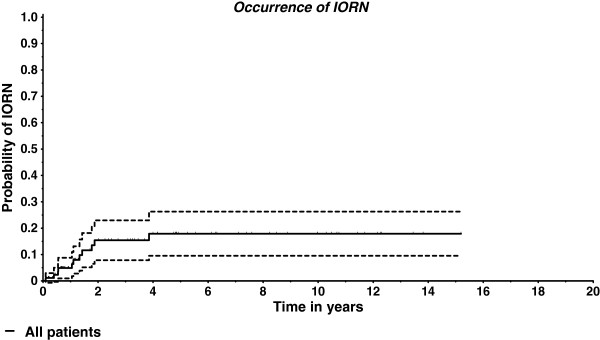
**Occurrence of IORN over time (Kaplan-Meier estimate and 95% confidence interval).**

In the group treated by hyperfractionation no patient with IORN could be identified. The Kaplan Meier estimate shows that IORN normally occurs within the first four years after radiotherapy, after that time new IORN cases were very rare (Figure 1).

All parameters mentioned in Tables 1, 2, 3 and 4 were tested univariately for potential prognostic significance for the occurrence of IORN. Results of this analysis are depicted in Table 5. The number of carious teeth and of odontogenic cysts in the lower jaw were found significant prognostic factors, for the numbers of present teeth before dental treatment and of remaining teeth after dental treatment a trend can be assumed. Multivariate analysis showed “odontogenic cysts” as the only significant factor.

**Table 5:** Prognostic factors for the occurrence of IORN in the lower jaw

**Univariate analysis**		
**Teeth**	**p=**	**Remarks**
*Dental status before starting radiotherapy*		
Absent	0.093	
Present	0.095	Trend
Carious	0.047	Significant
Destroyed	0.556	
Loose	0.730	
Root remainders	0.247	
Devital	0.688	
Roots – filled completely	0.555	
Roots – filled incompletely	0.774	
Apical periodontitis	0.956	
Cysts	0.023	Significant
Retained	0.291	
Conservative treatment possible	0.129	
No conservative treatment possible	0.830	
Filled	0.758	
Not sufficiently filled teeth	0.517	
Teeth with not sufficient crowns	0.897	
Item		
Chronic periodontitis with less to moderate attatchment loss	0.572	
Localized		
General		
Chronic periodontitis with severe attatchment loss	0.548	
Localized		
General		
Biofilm	0.188	
None		
Moderate		
Intense		
Dental calculus and subgingival concrements	0.188	
None		
Moderate		
Intense		
Partial prosthesis in lower jaw	0.5254	
Complete prosthesis in lower jaw	0.9026	
*Dental treatment before radiotherapy*		
Endodontic treatment	0.474	
Removal of root remainders	0.413	
Tooth extraction	0.939	
Conserving treatment	0.498	
Cystectomy	0.261	
Healthy teeth remaining after dental rehabilitation	0.085	Trend
Dental treatment after radiotherapy	0.768	
Demographic and oncological data		
Age	0.118	
Karnofsky performance status	0.455	
T-stage	0.784	
N-stage	O.797	
Total dose	0.774	
BED2	0.410	
Daily fraction	0.170	
**Multivariate analysis**		
Carious teeth	0.1808	
Present teeth	0.3154	
Odontogenic cysts	0.0180	Significant
Healthy teeth remaining after dental treatment	0.1552	

The treatment of infected osteoradionecrosis consisted of non-continuously bone resection in two patients, surgery without bone resection in further three. Two patients were treated conservatively while one underwent hyperbaric oxygen therapy. The remaining three patients were not treated at all.

### Oncological results

Due to the patients’ very limited compliance the outcome data were incomplete. The following numbers show the frequency of recurrence or progression compared to the number of patients with sufficient data available.

**Figure 2:**
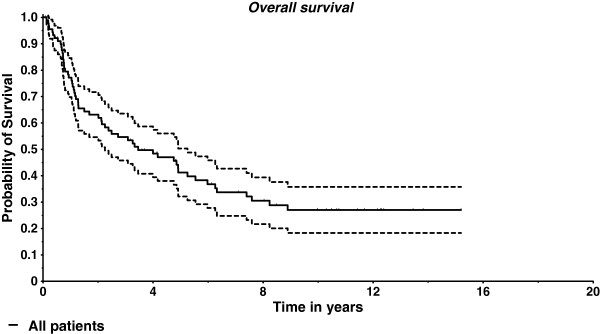
**Overall survival (Kaplan-Meier estimate and 95% confidence interval).**

A local tumor progression was found in 25/73 patients (34%), a progression in the regional lymph nodes in 12/61 patients (20%), and distant metastases in 12/59 patients (20%). No sufficient data to this point were available from the remaining patients. The median survival time was 3.1 years. The two-year survival rate was 62% while the five-year survival rate amounted to 41%. The Kaplan-Meier estimate for overall survival is depicted in Figure 2. As to acute side effects occurring during or shortly after radiotherapy, oral mucositis grade II WHO was found in 47 patients (54%), grades III and IV in further 5 patients (6%, n = 87). Sialadenosis (dryness of mouth) as a typical long-term side effect of radiotherapy was found in 72 patients (82%; grade 1 EORTC/RTOG: 22 patients (25%), grade 2: 38 patients (43%), grade 3: 12 patients (14%); n = 88).

## Discussion

The authors are well aware of the limitations of this retrospective evaluation. In this nearly homogenous collective of patients with oral cavity cancer having undergone radiotherapy +/- surgery, we have found complete data sets concerning dental status and restoration procedures of nearly all patients. The IORN data have been investigated meticulously, but due to the known incompliance of head and neck patients we cannot exclude that single events did not become known to the authors.

### Dental health status

The comparison of our data concerning dental findings before radiotherapy to those of the Forth German Trial of Oral Health (original name in German: IV. Deutsche Mundgesundheitsstudie des Instituts der Deutschen Zahnärzte DMS IV) resulted in notable differences [6]. Summarizing the data of more than 4000 Germans before dental treatment, in adults (33–44 years of age) on average 14.5 teeth were found carious, in older people (> = 45 years of age) 22.1 teeth. These teeth were rehabilitated completely in 95.6% and in 94.8%, respectively. A mean of 2.77 teeth in adults and of 14.2 teeth in older people were missing. 72% of the adults and 60.6% of the seniors were found to perform sufficient mouth hygiene. All these values were improved compared to the results of a former trial in 1997. On the other hand, the frequency of periodontitis was rising (moderate in 52.9% and intense in 39.8% of the population). Compared to those data our findings in patients with oral neoplasms are much more unfavourable. Further equally detailed analyses are rare. Jham et al. reported 2008 a collective of 207 patients with head and neck cancer with similar dental findings detecting periodontal disease in 41%, retained roots in 21%, carious teeth in 12%, and unerupted teeth in 5.8% of their patients, resulting in an IORN rate of 5.5% [7]. Schuurhuis et al. summarized 2011 the data of 185 patients and found oral infectious foci in 75%, a periodontal pocket depth of more than 6 mm in 23%, severe caries in 4%, impacted teeth in 4%, and residual root tips in 3%. Tooth extractions had to be performed in 30% of the patients, a mean of 7.7 teeth had to be removed. Periodontal treatment was performed in 6%. IORN was diagnosed in 11% [8]. Further literature data on this topic have been summarized in Table 6[7,9-12]. In general, tumor patients frequently show a noncompliance in routine dental care and daily oral hygiene. Tumor diagnosis did not change the patients’ habits: Lockhart and al. [12] stated that 97% of their patients needed dental care before radiotherapy, but only 81% underwent the indicated treatment.

**Table 6:** Dental status and rehabilitation procedures in the literature for IORN in the upper and lower jaw

**Author group**	**Year of publication**	**Dental status**	**Rehabilitation procedures**	**Remarks**
Frydrych et al. (n = 82) [9]	2011	No information	No information	Average (median) date of last dental visit: 66.76 months (18 months) before radiotherapy
Guggenheimer et al. (n = 947) [10]	1994	Edentulous: 59%	No information	
		Partially edentulous: 9%		
		Poor dentition with no replacement: 14%		
		Intact dentition: 18%		
Maier et al. (n = 100) [11]	1993	Tumour vs. control patients: Tartar > 3 mm: 40.91 vs. 21.98%	No information	Tumour vs. control patients
				Never tooth brushing 44.9 vs. 23.5%
		Decayed teeth >50% : 27.2 vs. 3.9%		Dental visit more than once a year: 6% vs. 43.5%
Lockhart et al. (n = 131) [12]	1994	Alveolar bone loss: 66%	Needing dental care: 97%	Noncompliant with routine dental care: 76%
		Clinical caries: 71%	Did not seek the indicated treatment: 81%	Noncompliant with routine oral hygiene: 65%
		Failing restorations: 91%		
Jham et al. (n = 207) [7]	2008	Periodontal disease: 41%	No information	
		Residual root: 21.2%		
		Caries 12%		
		Unerupted tooth: 5.8%		

We can summarize from this data that patients with head and neck neoplasms show a far more unfavorable denta health status than a healthy population does; our findings are well within the range of the data taken from the literature seen above.

### Frequency of IORN

The incidence of IORN varies widely (0 – 74%) as depicted in Table 7 whereas the majority of data are in a range of 5-10%. However, the comparison of these values to each other and to our results is very difficult due to a different definition or staging of IORN, different tumor localizations, therapy schedules, radiation techniques and dosages. Our results fit well within the range of data taken from the literature [5,7,13-29]. One of the data sets in the literature most comparable to our dataset has been published by Lee et al. [19] who experienced comparable IORN frequencies in a collective of patients having been operated on mainly.

**Table 7:** Incidence of IORN of the upper and lower jaw in the literature

**Author group**	**Year of publication**	**Incidence**	**Remarks**
Ben-David et al. [13] (n = 176)	2007	0	Multiple tumour localizations
			Primary treatment (no surgery)
			IMRT
			108/176 radiochemotherapy
Berger et al. [14]	2010	1-5%	Literature survey
Crombie et al. [15] (n = 54)	2012	36%	53/54 radiochemotherapy
Gomez et al. [16] (n = 168)	2011	1.2%	Multiple tumour localizations
			IMRT
Gomez et al. [29] (n = 35)	2009	5%	IMRT
Jerecek-Fosså et al. [17]	2002	0.4-56%	Literature survey
Jham et al. [7] (n = 207)	2008	5.5%	Head and neck cancer
Katsura et al. [18] (n = 39)	2008	15%	
Lee et al. [19] (n = 189)	2008	6.6%	Oral cavity and oropharynx
Monnier et al. [20] (n = 73)	2011	40%	Oral cavity and oropharynx
Oh et al. [21] (n = 81)	2004	4.9%	
Reuther et al. [5] (n = 830)	2003	8.2%	Oral cavity and oropharynx
Stenson et al. [22] (n = 27)	2010	18.4%	Surgery, adjuvant radiochemotherapy
Storey et al. [23] (n = 83)	2001	6%	Malignant submandibular tumours
Studer et al. [24] (n = 304)	2011	Grade 2 EORTC: 1.6%	Oral cavity and oropharynx
			Conventional dental care vs. risk-adapted dental care
			IMRT
Thiel [25]	1989	4-35%	Literature survey
Thorn et al. [26] (n = 80)	2000	74%/3 years	Multiple tumour localizations
Tsai et al. [27] (n = 402)	2013	7.5%	Oropharyngeal cancer, median time to IORN 8 months
Turner et al. [28] (n = 333)	1996	5.9%	

### Risk factors for the development of IORN

Numerous prognostic factors for the development of IORN have been tested and published. A selection of these is summarized in Table 8[3,4,14,17,18,20,27,28],[30-39]. The localization of the primary tumour in the oral cavity with its microbial colonization and the abundant involvement of the mandibular bone with its unique blood supply probably promotes IORN. Unfavourable dental status, periodontal disease and irritation of the gingiva by pressure sore triggered by dental prosthesis are important as well as dental extractions before and especially after radiotherapy and do also promote an infestation of the upper jaw.

**Table 8:** Risk factors for IORN of the upper and lower jaw in the literature

**Author group**	**Year of publication**	**Risk factor(s)**	**Remarks**
Ahmed et al. [30]	2009	Intensity modulated radiotherapy (IMRT) advantageous compared to conventional radiotherapy	
Berger et al. [14]	2010	Total dose >66Gy	Literature survey
Bhide et al. [31]	2012	Total dose > 60Gy	Literature survey
		Volume of mandible within the treatment field. Trauma related ORN after lower doses	
		IMRT	
Chopra et al. [32]	2011	White ethnicity	
		Secondary infection	
		Advanced age	
		Stage IV	
		Total dose	
		Post-RT dental extractions	
		Lack of pre-RT dental extractions	
Goldwasser et al. [33]	2007	Higher body mass index	Multivariate analysis
		Use of steroids	
		Radiation dose >66Gy	
Jerecek-Fosså et al. [17]	2002	Total dose	Literature survey, only part of the factions mentioned in the paper cited here
		Brachytherapy dose	
		Dose per fraction	
		Interval between fractions	
		Volume of the horizontal ramus of the mandible irradiated with a high dose	
		Dental status	
		Bad oral hygiene	
		Dental extractions after radiotherapy	
Katsura et al. [18]	2008	Oral health status after radiotherapy	
		Periodontal pocket depth	
		Dental plaque	
		Alveolar bone loss level	
		Radiographic periodontal status	
Lee et al. [19]	2009	Univariate: Mandibular surgery	Multivariate analysis: Mandibular surgery
		Co-60	BED >106.2Gy
Lozza et al. [35]	1997	Dose rate	Brachytherapy exclusively
		Reference volume	
Curi et al. [3]	1997	Oral cancer	
		Invasion of bone	
		Tumour surgery	
		Total radiation dose	
		Dose rate/day	
		Mode of radiation delivery	
		Dental status	
		Time from radiation therapy until the onset of ORN	
Monnier et al. [20]	2011	Oral cavity tumours	Multivariate analysis: bone surgery
		Bone invasion	
		Surgery prior to radiotherapy	
		Bone surgery	
Nabil et al. [36]	2012	Hyperfractionation	Literature survey
		Reduced risk after accelerated radiotherapy with reduced dose	
Reuther et al. [4]	2003	Advanced tumours	
		Segmental resection of the mandible	
		Tooth extractions (pre/post RT)	
		Pre-surgical radiotherapy worse than post-surgical radiotherapy	
Støre and Boysen [37]	2000	Tumour localization in tongue and floor of mouth	
		trauma	
Thiel et al. [38]	1989	Caries	
		Periondontosis	
		Periapical pathology	
		Injury	
		Irritation by prostheses	
		Dental extractions before and after radiotherapy	
		Bone surgery because of remaining or recurrent tumours	
Thorn et al. [39]	2000	Removal of teeth	
		Surgery	
		Injury from prosthesis	
		Spontaneous breakdowns	
Tsai et al. [27]	2013	Total dose	
		Dental status	
		Smokers	
		Alcohol	
		Larger tumours	
Turner et al. [24]	1996	Bone involvement	
		Synchronous Methotrexate	
		Scattered dose from elective	
		neck treatment	
		Increasing dose	
		Increasing target volumes for doses <55Gy	
		Dental extractions	

Radiation dose should not exceed 60 – 66Gy to the mandibular bone whenever possible, the target volume within the bone should be limited. Some authors regard hyperfractionation a risk factor for IORN whereas Intensity modulated radiotherapy (IMRT) has been found advantageous compared to conventional 3D-planned radiotherapy. Additional factors may be chemotherapy, higher body mass index and the use of steroids.

We found odontogenic cysts and carious teeth to be significant prognostic factors univariately and odontogenic cysts multivariately. This rather unusual finding may result from the low number of patients in the IORN group and the fact that for some variables the data were incomplete.

Apart from this analysis, hyperfractionation seemed to have a protective effect whereas this could not be examined further due to the small number of events. In our ancient publication on this topic [2] (a very detailed comparison is currently under preparation) we experienced a very high frequency of IORN after hyperfractionated radiotherapy which may have been caused by to high total doses on the one hand and a too short interfraction interval (time interval between the two daily fractions) on the other hand. Both factors have been taken into account here, consequently the results were improved markedly.

An important paper has been published by Tsai et al. in 2013 [27]. They reviewed the records of patients with small oropharyngeal cancers having undergone radiotherapy or radiochemotherapy. The overall prevalence of IORN was 7.5%, higher doses, use of nicotine and alcohol, dental status as well as more advanced tumors were found significant risk factors for the development of IORT. In contrast to this paper our patients’ primary situation seems more unfavorable: we only examined patients with oral cancer where the whole mandible was within the 100%-isodose, thus we applied even higher doses to a large amount of bone. Furthermore, older techniques have been used; unfortunately, no information about fractionation has been given. Consequently, a higher prevalence of IORN here seems to be explainable.

## Conclusions

In our patient collective the dental status was very poor, extensive dental and oral and maxillofacial restoration procedures had to be performed. This meticulous dental care resulted in an incidence of IORN of 12%, all of them had undergone conventionally fractionated radiotherapy. It was very interesting to see that in the hyperfractionated group no IORN occurred at all. Significant prognostic factors could not be found. Our data fit well to those taken from the literature.

## Abbreviations

ORN: Osteoradionecrosis; IORN: Infected osteoradionecrosis; RT: Radiotherapy; RCT: Radiochemotherapy; WHO: World Health Organization; EORTC: European Organization for research and treatment of cancer; RTOG: Radiation therapy oncology group; DMS: Forth German trial of oral health; ICRU: International commission on radiation units.

## Competing interests

The authors declare that they have no competing interests.

## Authors’ contributions

MN supervised data acquisition and evaluation and wrote the manuscript. JF was responsible for the patients’ radiotherapy procedures and revised the manuscript. KM collected the data and re-examined the x-rays. The data presented here are a part of Mrs. M’s thesis. HH and WJS revised the manuscript in the fields of dentistry and oral and maxillofacial surgery. CR supervised the patients’ radiotherapy and approved the manuscript. All authors read and approved the final manuscript.
